# LeukoCatch, a quick and efficient tool for the preparation of leukocyte extracts from blood

**DOI:** 10.1186/1472-6890-11-9

**Published:** 2011-08-17

**Authors:** Daisuke Okuzaki, Shoichi Kimura, Norikazu Yabuta, Toshinari Ohmine, Hiroshi Nojima

**Affiliations:** 1 Department of Molecular Genetics, Research Institute for Microbial Diseases, Osaka University, 3-1 Yamadaoka, Suita, Osaka 565-0871, Japan; 2 DNA-chip Development Center for Infectious Diseases, Research Institute for Microbial Diseases, Osaka University, 3-1 Yamadaoka, Suita, Osaka 565-0871, Japan; 3 Fukae Kasei Co., Ltd., 3-2-44 Takatsuka-dai, Nishi-ku, Kobe, Hyogo 651-2271, Japan; 4 Laboratory for Clinical Investigation, Osaka University Hospital, Osaka University, 2-15 Yamadaoka, Suita, Osaka 565-0871, Japan

**Keywords:** cell extract, leukocyte, diagnosis, PBMC, proteomics

## Abstract

**Background:**

Whole-protein extracts from peripheral blood leukocytes are ideal for basic and clinical research. However, lack of a simple preparation technique has limited the use of such extracts. The aim of this study is to develop a simple and easy system that can selectively obtain leukocyte extracts without hemoglobin.

**Methods:**

A filter that captures the leukocytes but not RBCs was set at the bottom of a 10-mL medical syringe by sandwiching it between plastic stoppers. The capturing efficiency of leukocytes with this tool, called LeukoCatch, was examined using human macrophage cells (MONO-MAC-6). The abilities of LeukoCatch system to capture the leukocyte proteins and to remove the hemoglobin from RBCs were tested by western blot analysis using human blood samples.

**Results:**

This study presents the development of LeukoCatch, a novel tool that allows the preparation of leukocyte extracts from blood samples within 3 min without centrifugation. Tissue-cultured human macrophage cells were tested to determine the optimal filter numbers and pass-through frequencies of LeukoCatch, which was then applied to 2-mL blood samples. Samples were passed 2~5 times through a LeukoCatch equipped with 5 filters, washed twice with phosphate-buffered saline for red cell removal, and leukocyte proteins were extracted with 0.5 mL of elution buffer. Western blot analysis of the purified extract indicated that more than 90% of hemoglobin was removed by the LeukoCatch and that the protein recovery rate of leukocytes was at least 4 times better than that of the conventional centrifugation method.

**Conclusion:**

We conclude that LeukoCatch is useful not only for diagnosis at the bedside but also for basic research using blood samples or tissue culture cells.

## Background

Peripheral blood is an ideal surrogate tissue for disease diagnosis and prognosis. Extensive data have been accumulated from the genome-wide gene-expression profiling of patient peripheral blood mononuclear cells (PBMCs) and their leukocyte subpopulations using DNA microarray analyses [1,2] in a wide range of diseases, including cancer [3,4] and autoimmune diseases [5]. Moreover, novel approaches or revolutionary tools for transcriptomics are rapidly emerging [6]. These data reveal the up- or down-regulated genes that can serve as potential RNA diagnostic markers of relevant diseases [7-10]. If the marker gene products (mostly proteins) could be detected by antibodies using the extracts of leukocyte subpopulations in the PBMCs, the transciptome data would be practically useful for bedside diagnosis. Generally, the instrumentation required for RNA diagnostics is not routinely available at the bedside or in a common clinical facility.

Most antibody-based diagnostics target proteins secreted into the serum or protruding from the cell membrane surface. Such targets are easily detected by specific antibodies via enzyme-linked immunosorbent assay (ELISA) or dot blot analysis using the supernatant or sediments of the blood sample. To efficiently analyze leukocyte protein extracts for expression profiling by specific antibodies, the white blood cells (WBCs) or leukocytes must be separated from the red blood cells (RBCs) that normally occupy about half of the total blood volume. Red blood cells contain abundant iron-rich hemoglobin, which can hamper the analysis of the comparatively small amount of leukocyte proteins.

The separation of RBCs from leukocytes has been primarily based on methodology established through the pioneering work of Bøyum [11]. Separation media consisting of a mixture of Ficoll 400 (or Percoll) and an iodinated density gradient medium have been very widely used to purify human leukocytes in basic research and in routine diagnostic studies. Nevertheless, variable extract recovery rates and/or possible changes in the expression profiles during the several-hour separation procedure can disturb the data reproducibility. Moreover, the leukocyte separation procedure requires an expensive centrifugation facility that is not routinely available at the bedside. Thus, a quick, easy, inexpensive, and efficient method for the purification of leukocyte extracts is needed.

In the present study, we present LeukoCatch, a novel tool for the centrifugation-free purification of leukocyte extracts from whole blood. LeukoCatch consists of a layer of filters held at the bottom of a syringe, which captures leukocytes but not RBCs from a blood sample. Captured leukocytes are washed by phosphate-buffered saline (PBS) and the protein extract of leukocytes were collected as the flow-through fraction that was obtained by flushing the filter with extraction buffer. Western blot analyses revealed that the collected extracts lacked most RBC components and retained a sufficient amount of leukocyte-derived proteins. We propose that LeukoCatch is useful not only for diagnosis at the bedside but also for basic research.

## Results

### Structure of LeukoCatch and its usage

**Figure 1:**
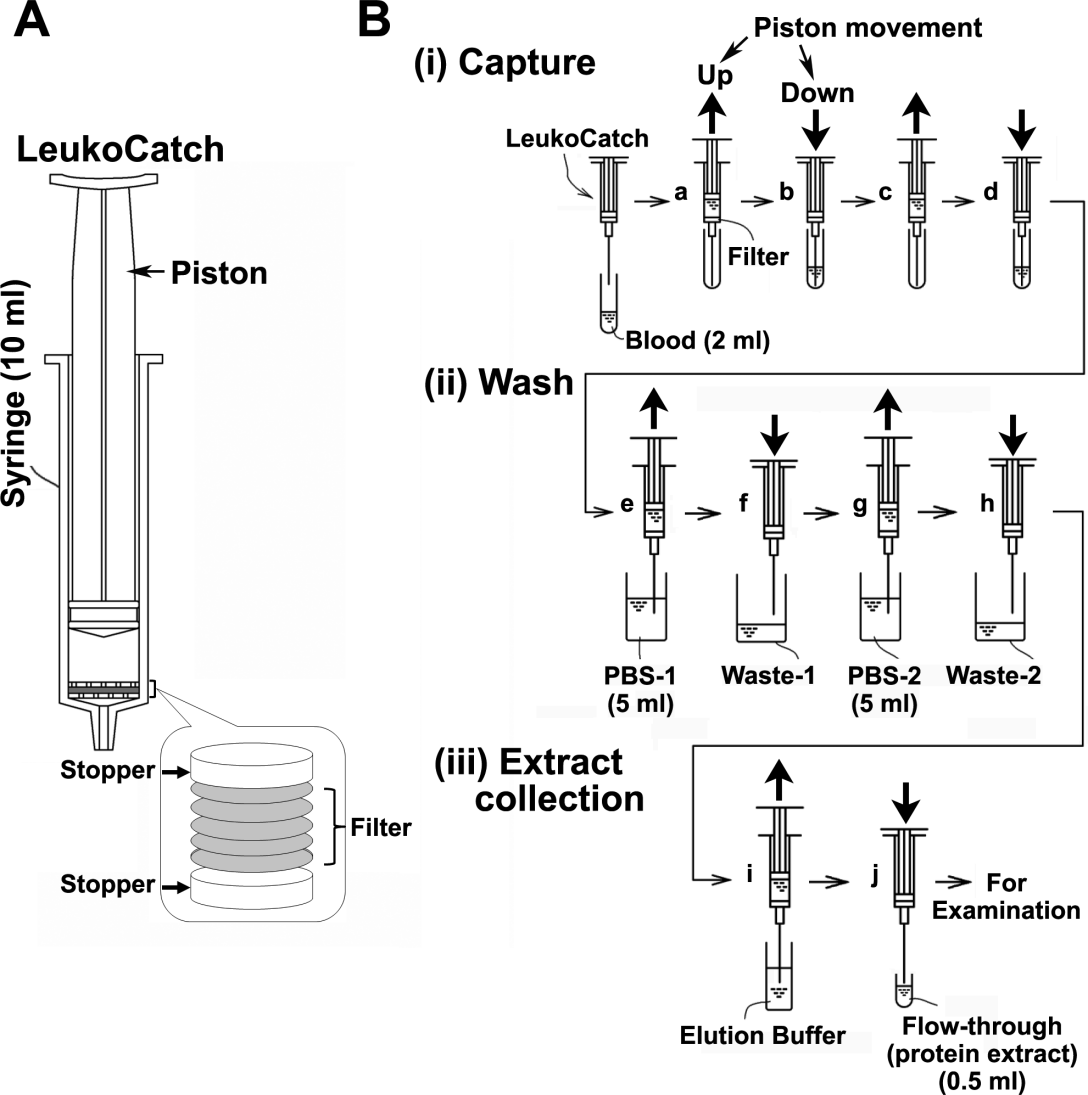
**Schematic drawings of the LeukoCatch system and its usage**. (A) Structure of the LeukoCatch system in which the stacked filters are installed between the stoppers at the bottom of a commercially available syringe. An enlarged view of the stacked configuration of the filtering system is shown in an inset. (B) A schematic drawing for a protocol for preparation of leukocyte (or PBMC) extract from 2 ml blood sample. The protocol consists of three procedures: capture of leukocyte by the LeukoCatch filters (i), leukocyte wash by PBS (ii), and collection of leukocyte extract in the elution buffer of the flow-through. (iii) The leukocyte extract in the flow-through fraction is ready for examination (e.g., by western blot analysis or ELISA). Thin horizontal arrows show the flow of the procedure (from step **a**, to step **j**). Thick vertical arrows indicate the multiple movements of the piston to allow the blood (i), PBS (ii) or elution buffer (iii) pass through the stacked filters in the LeukoCatch system. In each step, the piston was moved up (steps **a, c, e, g **and **i**) or down (steps **b, d, f, h **and **i**) by hand. The whole procedure may be completed within 3 min.

Since RBC hemoglobin and serum immunoglobulin comprise most of the protein in blood cells, they must be removed to analyze the variation of protein components in leukocytes. A LeukoCatch filter (Pall, Leukosorb B Medium, LKB-3R) was set at the bottom of a 10-mL medical syringe by sandwiching it between plastic stoppers (Figure 1A). This filter is expected to capture the leukocytes but not RBCs or platelets, and has been successfully used at the bedside (Leukotrap filter; see http://www.pall.com/medical_39479.asp) to prepare leukocyte-depleted platelet concentrates from whole blood [12-14]. We surmised that the leukocytes but not RBCs of the peripheral blood would be efficiently captured by passing through a small number of layered filters (Figure 1B-i) by moving the piton up (steps **a **and **c**) and down (steps **b **and **d**) by hand, and that the hemoglobin and immunoglobulin would be removed by washing the filter with PBS (Figure 1B-ii) with upward (steps **e **and **g**) and downward (steps **f **and **h**) piton movement. The whole-protein extract, collected by rinsing the filter with EB by upward (step **i**) and downward (step **j**) piton handling, would be ready for further examination by western or dot-blot analysis (Figure 1B-iii).

### Optimal filter number in the LeukoCatch to capture macrophage cells

**Figure 2:**
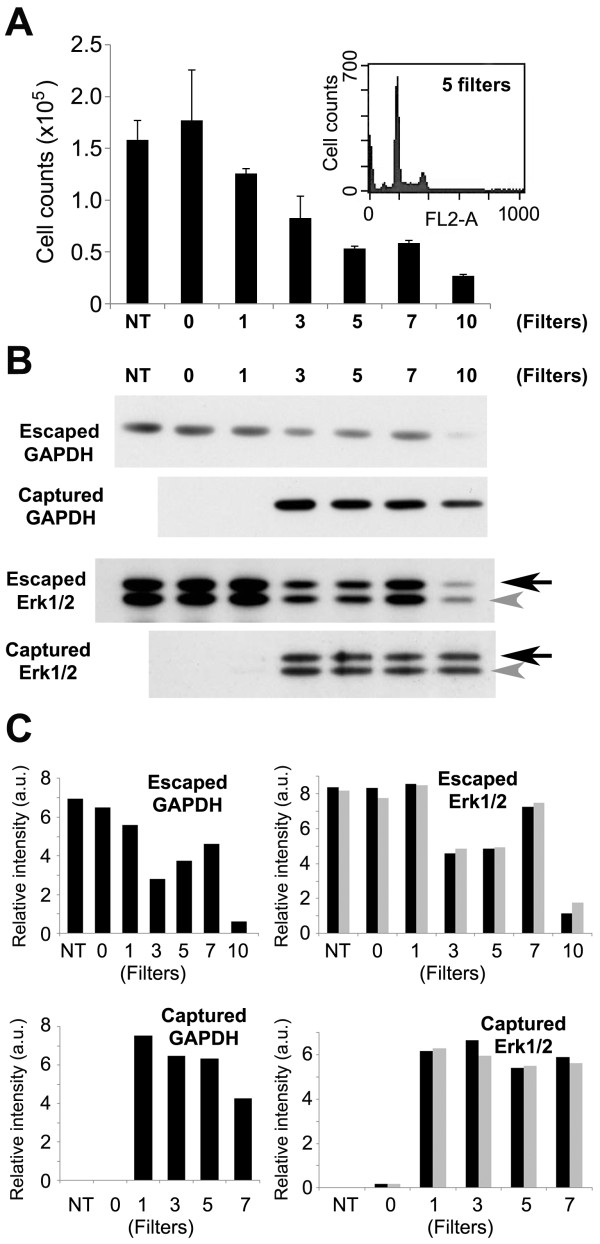
**Effect of the filter number on macrophage capture by the LeukoCatch**. (A) Human macrophage (MONO-MAC-6) cells were examined as a surrogate of leukocytes in the blood. Cells were subjected to serial passage (twice) of the same syringe (LeukoCatch system) that was equipped with 0, 1, 3, 5, 7 or 10 filters. NT signifies the non-treated blood sample. The "0" differs from NT because in "0", the blood sample was passed through the LeukoCatch system with a zero filer. The bar graphs represent the number of macrophage cells (flow-through) that were not captured with the filters. Cell counts were obtained by FACS (n = 4). (B) Western blot analysis by anti-GAPDH and anti-Erk1/2 antibodies using a 5-μL extract (10% of total) of cells escaped from or captured by the indicated numbers of filters in the LeukoCatch. (C) The bar graphs represent the relative intensity of the band signals measured by using the Image-J software, which were given as percentage of the starting material (NT: non-treated).

To determine the optimal number of filters in the LeukoCatch, we examined the capturing efficiency of human macrophage cells (MONO-MAC-6). When the number of filters was varied from zero (NT: non-treated) to ten and the number of captured cells was counted by FACS, > 60% of cells were captured by a LeukoCatch equipped with 5, 7, or 10 filters after taking in and expelling the MONO-MAC-6 cells in the medium once (Figure 2A).

Western blot analysis was performed using antibodies for GAPDH and Erk1/2, which are expressed by MONO-MAC-6 cells, using a 5-μL extract (10% of total) of escaped cells. The amount of passed-through proteins was reduced relative to the number of filters used, as judged by direct observation (Figure 2B) or quantification of the band intensities by using the Image-J software (Figure 2C). Similar western analysis using a 5-μL extract (10% of total) of captured cells indicated that the LeukoCatch with > 3 filters was practically useful for capturing these proteins. The band intensities were saturated due to abundant captured proteins when 3 to 10 filters were employed, revealing the excellent capture efficiency of the LeukoCatch. Since passing the sample through 7 or 10 filters is practically difficult (due to the sticky movement of the syringe plunger), we used 5 filters (i.e., a "5-filtered LeukoCatch") for further experiments.

### Optimal passage frequency through the LeukoCatch for macrophage cells

**Figure 3:**
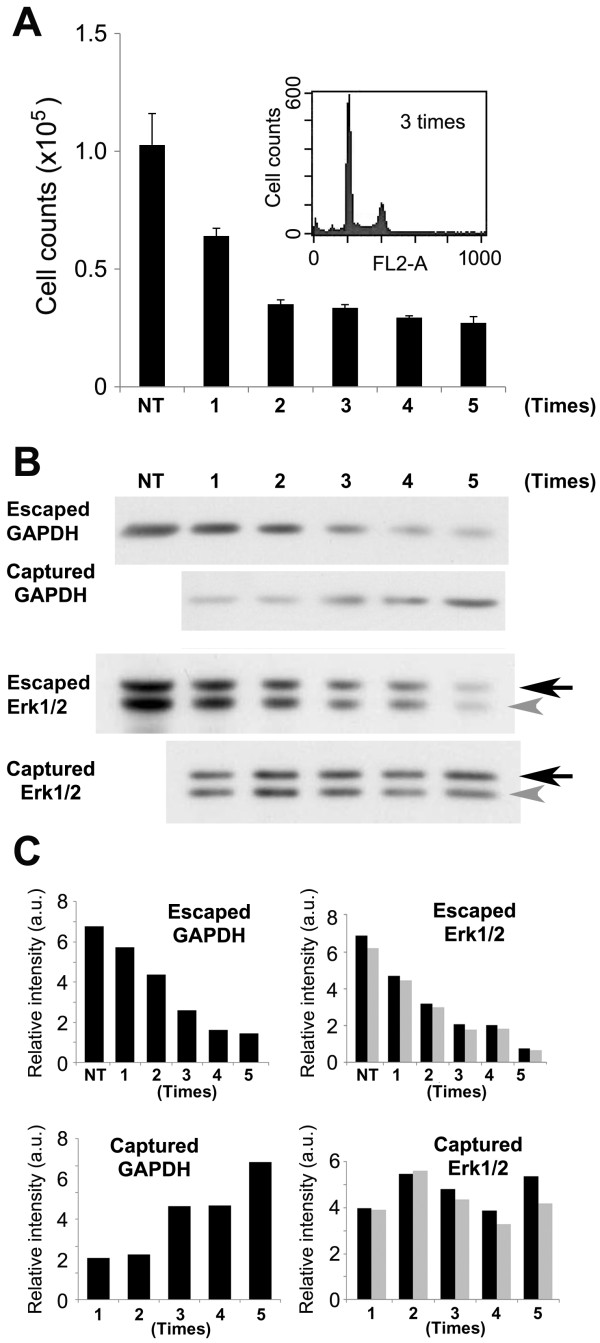
**Effect of the passage frequency on macrophage capture by LeukoCatch**. (A) Human macrophage (MONO-MAC-6) cells were subjected to serial passage (1, 2, 3, 4 or 5 times) of the same syringe (LeukoCatch system) with a stack of 5 filters. NT, non-treated. The bar graphs represent the number of macrophage cells (flow-through) that were not captured with the LeukoCatch system after serial passage. Cell numbers were counted by FACS (n = 4). (B) Western blot analysis with anti-GAPDH and anti-Erk1/2 antibodies using a 5-μL extract (10% of total) of cells that escaped from or were captured by the LeukoCatch filters, after being passed through the indicated number of times. (C) The bar graphs represent the relative intensity of the band signals measured by using the Image-J software, which were given as percentage of the starting material (NT: non-treated).

We next varied the number of times the MONO-MAC-6 cells were passed through the 5-filtered LeukoCatch (i.e., the "passage frequency"). FACS analysis indicated that 2-5 passages effectively captured > 60% of cells. Western analysis for both GAPDH and Erk1/2 indicated that the amount of passed through (escaped) proteins was reduced relative to the passage frequency through the 5-filtered LeukoCatch (Figure 3A). In contrast, the ratio of the captured GAPDH increased relative to the passage frequency (Figure 3B). The amount of captured Erk1/2 was unaltered, probably due to saturation since Erk1/2 is abundant in MONO-MAC-6 cells. These results indicate that use of ≥ 5 passes through the 5-filtered LeukoCatch is preferable.

### Effect of filter number and passage frequency on leukocyte capture from blood samples

**Figure 4:**
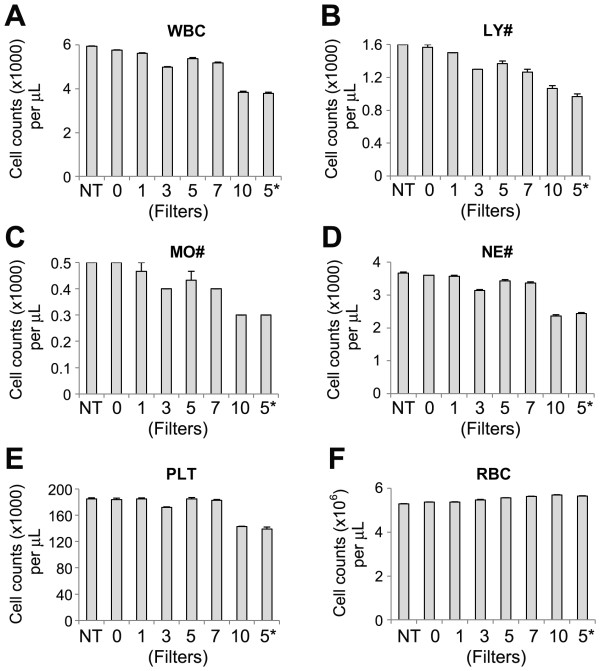
**Effect of the filter number on the capture of PBMCs by the LeukoCatch**. Indicated blood cells were counted by the Coulter LH750 hematology analyzer, using human peripheral blood samples (N = 3) passed once through the LeukoCatch equipped with 0-10 filters. In the 5 × 5 sample, the blood sample was passed 5 times through a 5-filtered LeukoCatch. NT, non-treated blood samples; WBC, white blood cell; LY#, lymphocyte number; MO#, monocyte number; NE#, neutrophil number; PLT, platelet; and RBC, red blood cell.

To assess the optimal number of filters in the LeukoCatch for blood samples, we examined the leukocyte capturing efficiency using fresh human blood. Hematologic analysis of the blood samples was performed immediately after passing each sample once through the LeukoCatch equipped with various numbers of filters. The number of passed-through WBCs, leukocytes, and other leukocyte subpopulations decreased with an increasing number of filters (Figure 4A). Notably, when the blood was passed through 5 times with the 5-filtered LeukoCatch, we obtained a similarly excellent efficiency as was found with the 10-filtered LeukoCatch. Similar capture efficiencies were obtained for the lymphocyte (LY), monocyte (MO), neutrophil (NE), and PLT subpopulations (Figure 4B-E). No RBCs appeared to be captured by the LeukoCatch (Figure 4F). Slight increase in the number of RBCs is probably due to the fragmentation, namely, the RBCs fragmented during the passage of the filters were counted as independent RBCs by the hematology analyzer used in this experiment. These results indicate that leukocyte cells are efficiently captured by the 5-filtered LeukoCatch.

**Figure 5:**
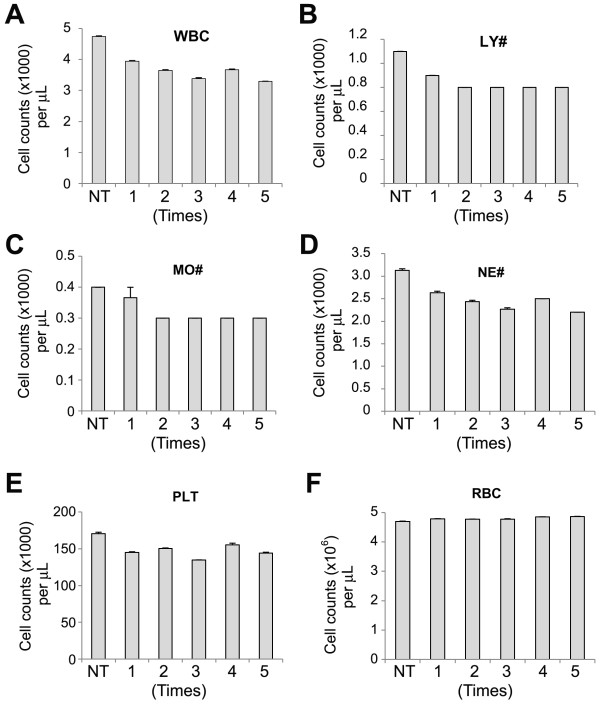
**Effect of the passage frequency on the capture of PBMCs from blood through the LeukoCatch**. Indicated blood cells were counted using human peripheral blood samples (N = 3) that were passed 1-5 times through a 5-filtered LeukoCatch. NT, non-treated blood samples; WBC, white blood cell; LY#, lymphocyte number; MO#, monocyte number; NE#, neutrophil number; PLT, platelet; and RBC, red blood cell.

We next varied the passage frequencies of blood samples through the 5-filtered LeukoCatch. Leukocytes were captured more efficiently with an increasing passage frequency (Figure 5A). Similar results were obtained for other leukocytes and PLTs, although some of their efficiencies appeared to be saturated, probably because of the limited numbers of their populations (Figure 5C-E). In contrast, no RBCs were captured even by passing 5 times through the 5-filtered LeukoCatch (Figure 5F).

### Western blot analysis to assess the leukocyte capture efficiency

To assess the extent of protein capture by the 5-filtered LeukoCatch system for blood samples, we performed western blot analysis for the proteins retained on the filter (retentate) after PBS wash, and then eluted into the flow-through fraction (Figure 1b, step j). We used anti-GAPDH and -Erk1/2 antibodies to detect proteins that exist in both leukocytes and RBCs. We also used antibodies to detect α-tubulin (expressed predominantly in leukocytes but less abundantly in RBCs), hemoglobin (expressed exclusively in RBC) and Ficolin 1 (FCN1: expressed exclusively in leukocytes; 15). To examine if the leukocytes harboring the membrane proteins may stick to filter fiber surfaces differentially and retained by the filters, we also tested antibodies against leukocyte specific membrane proteins such as CD14 and CD11a. FCN1 is a soluble collagen-like protein that binds sugar structures or acetylated compounds present on microorganisms and initiates activation of the lectin complement pathway [15]. CD14 is a component of the innate immune system that is either anchored into the leukocyte membrane or in a soluble form [16]. CD11a is a component of the Beta2-integrin CD11a/CD18 (LFA-1) that is a leukocyte transmembrane protein [17].

**Figure 6:**
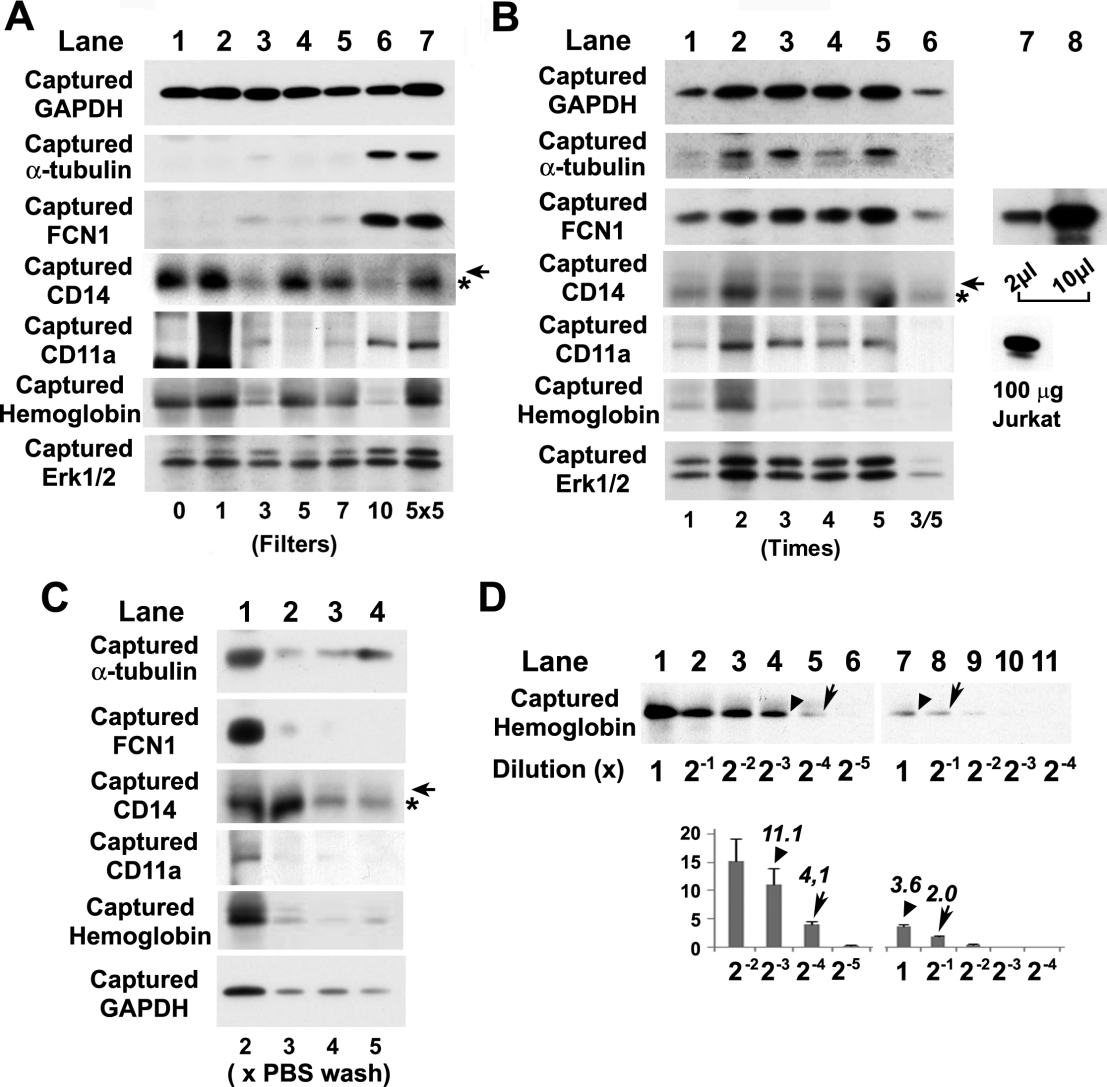
**Western blot analysis of captured PBMC extracts using anti-GAPDH, -α-tubulin, -FCN1, -CD14**, -**CD11a, -hemoglobin and -Erk1/2 antibodies**. (A) LeukoCatch was equipped with various numbers of filters. Analyzed samples were 10-μL extracts (from 40 μL of blood) of PBMCs captured with the indicated numbers of filters in the LeukoCatch. In the 5 × 5 sample, the blood sample was passed 5 times through a 5-filtered LeukoCatch. Horizontal arrow or asterisk indicates the band for CD14 or immunoglobulin, respectively. (B) Passage frequency of the blood sample through the LeukoCatch was varied. Analyzed samples were 10-μL extracts of cells captured from the indicated number of passages through the LeukoCatch filters. Lanes 1-5 reflect blood washed twice with PBS (see steps **e **to **h **in Figure 1B). Lane 6 (sample 3-5) reflects blood washed 5 times with PBS. In lanes 7 or 8, a PBMC extract (2 μL from 160 μL blood or 10 μL from 0.8 mL blood) prepared by the standard method for PBMC preparation using centrifugation in the presence of Ficoll [11] was analyzed. As for CD11a, 100 μg extract of Jurkat cells was also examined using the same anti-CD11a antibody to confirm the identity of detected bands. (C) The number of PBS washes was varied. Analyzed samples were 10-μL extracts of cells captured by the LeukoCatch after the indicated number of PBS washes. (D) Western blots for estimation of the removed red blood cells from the filters by monitoring the captured hemoglobin. The leukocyte extract obtained by the serial two-fold dilution of non-treated blood samples (lanes 1-6) or the leukocyte extract using the 5-filtered LeukoCatch (lanes 7-11) were subjected to western blot and probed with anti-hemoglobin antibody. The bar graph in the lower panel shows the intensity of each band measured by using the Image-J software. Numbers above the bars represent the mean values of band intensities from three independent films of distinct exposure time of the western blots (1 min, 10 sec and 1 sec). Pairs of arrows or arrowheads represents the compared bands (see text).

We found that α-tubulin, FCN1, CD14, and CD11a showed strong bands only when samples were passed once through the 10-filtered LeukoCatch (Figure 6A, lane 6) or 5 times through the 5-filtered LeukoCatch (Figure 6A, lane 7), while GAPDH and Erk1/2 served as loading controls (Figure 6A lanes 1-7). Thus, not only the leukocyte soluble protein (FCN1) but also the leukocyte membrane proteins (CD14 and CD11a) are recovered into the collected samples, suggesting that even a population of leukocytes carrying membrane proteins do not stick to filter fiber surfaces differentially and not retained by the filters. Notably, little hemoglobin was left in the 10-filtered LeukoCatch (Figure 6A, lane 6).

As expected from the results shown in Figure 5A, the capture efficiency of these proteins was increased when the sample was passed through twice rather than once (Figure 6B, lanes 1, 2), but similar results were obtained for passage frequencies of 2~5 times (Figure 6B, lanes 2-5). Notably, after washing with PBS 5 times for a 3-times passages through 5-filtered LeukoCatch (denoted by 3/5), the intensities of the bands for all proteins were dramatically reduced (Figure 6B, lane 6). Hemoglobin apperared to be removed during these repeated passages (Figure 6B, lanes 3-6).

When the PBS wash frequency was 2~5 times, the intensity of the FCN1 and hemoglobin bands were drastically reduced after 3~5 PBS washes (Figure 6C). These results indicate that the best protocol for the practical use of the LeukoCatch system is as follows; repeated (5 x) blood passages through a 5-filtered LeukoCatch with two PBS washes.

We compared these results with those of an extract prepared according to the conventional centrifugation of a blood sample on Percoll. A 10-μL extract derived from 40 μL of blood using the LeukoCatch showed an FCN1 band of similar intensity as that of a 2-μL extract derived from 160 μL of blood using the centrifugation method (Figure 6B, lanes 7 and 8). This result suggests that the protein recovery of the 5-filtered LeukoCatch was at least 4 times better than that of the conventional centrifugation method.

We also compared the amount of captured hemoglobin in the LeukoCatch without filters (Figure 6D, lanes 1~6) and with five filters (Figure 6D, lanes 7~11). We found that the band intensities of 2^-4 ^dilution (4.1, arrow) of the former was similar to 2^-0 ^(= 1) dilution (3.6, arrowhead) of the latter. This indicates that about 2^-4 ^or 1/16 dilution of hemoglobin was attained by the LeukoCatch, namely, more than 90% of hemoglobin was removed by the LeukoCatch. Comparison of the intensity of other bands (arrows or arrowheads) also supports this conclusion.

## Discussion

The present study reports a quick, easy, efficient, and centrifugation-free purification system of leukocyte extracts from PBMCs, using a novel tool containing layers of Pall Leukotrap filters (Figure 1). The Pall Leukotrap RBC inline filter system [12] permits the near-complete depletion of leukocytes from blood [14]. This filter system has been successfully applied to the bedside preparation of leukocyte-depleted platelet concentrates from whole blood or to remove exogenous infectious prions and endogenous infectious agents from red cell concentrates [18,19]. In the present study, the usefulness of this filter was extended to the preparation of whole cell extracts from tissue culture cells or leukocytes from blood samples.

We first developed a novel tool called LeukoCatch that consists of a layer of filters held at the bottom of a syringe to capture leukocytes but not RBCs from a blood sample (Figure 1). Using human macrophage (MONO-MAC-6) cells, we determined the optimal filter numbers (Figure 2) and passage frequencies (Figure 3) for the LeukoCatch system. The system was then applied to human blood. The most practical protocol involved 2~5 passages through the 5-filtered LeukoCatch (Figure 1) with two PBS washes, followed by protein extraction with EB (Figures 4, 5 and 6). Western blot analysis probed by a typical leukocyte protein FCN1 showed that the 5-filtered LeukoCatch showed at least 4 times better recovery rate of leukocytes than the sample prepared by conventional centrifugation of a blood sample on Percoll (Figure 6B).

Although the Leukocatch system removes 90% of hemoglobin (Figure 6D), it is still be a huge contaminant in these preparations, considering that hemoglobin in RBC is at a concentration of 5 mM and about half the volume of whole blood is RBCs. However, western blot analysis using typical leukocyte proteins displayed sharp bands without distortion of their shapes (Figure 6A-C). Without this system, western blot analysis using the whole blood would not be practical because hemoglobin represents the major part of the total protein and hampers the detection of other leukocyte proteins.

Notably, these leukocyte membrane proteins (CD14 and CD11a) were also recovered into the collected samples, suggesting that even a population of leukocytes carrying membrane proteins is not retained by the filters. To increase the efficiency of present 30-40% (Figure 4 and 5), we can increase the number of stacked filters or increase the volume of syringe. However, this is practically unnecessary because the present system already can provide the enough amount of leukocyte extracts for 50 times western blots for typical leukocyte proteins FCN1, CD14 and CD11a from 2 mL blood sample, considering the results shown in Figure 6B obtained by using 40 μL of blood samples (40 μL × 50 = 2mL).

Since the LeukoCatch system is simple, an automatic machine may be easily developed for the large-scale preparation of protein extracts from blood samples. Elution of the trapped lymphocytes by flushing EB into the Leukotrap filters (which are commonly discarded after bedside use) may yield useful lymphocyte extracts for basic and clinical research. Since functional human lymphocytes can be recovered from Leukotrap filters by back-flushing with cold PBS [20], LeukoCatch could also be useful as a quick and easy tool for the recovery of live lymphocytes.

## Conclusion

Taken together, we conclude that LeukoCatch is useful not only for diagnosis at the bedside but also for basic research using blood samples or tissue culture cells. Notably, the protein recovery of the LeukoCatch was at least 4 times better than that of the conventional centrifugation method, which indicates the advantage of this technique.

## Methods

### Analysis with MONO-MAC-6 cells

MONO-MAC-6 cells (DSMZ, Germany) were maintained in RPMI1640 medium supplemented with 20% fetal bovine serum (FBS, HyClone, Logan, UT), 10 μg/mL human insulin, 2 mM L-glutamine, 1 mM sodium pyruvate, 100 U/mL penicillin, and 100 μg/mL streptomycin in a CO_2 _(5%) incubator at 37°C. Cells in the logarithmic growth phase were used for experiments.

MONO-MAC-6 cells (2 × 10^6 ^cells/experiment) suspended in 5 mL of medium were passed through (sucked-and-poured) twice by the LeukoCatch, and then were divided into two tubes. One tube was subjected to FACS analysis. The other tube was centrifuged twice at 190 × *g *for 5 min each at 25°C, with the pellet washed with 2.5 mL PBS between centrifugations. After removing the supernatant, 25 μL of elution buffer (EB) [10 mM Tris-HCl (pH 7.5), 100 mM NaCl, 1% TritonX-100, 1 mM EDTA (pH 8.0), 0.1 mg/mL PMSF, 1 mM Aprotinin, 0.001 mg/mL Leupeptin, 0.001 mg/mL pepstatin A, 1 mM NaF, 1 mM Na_3_VO_4_, and 10 mM β-glycerophosphate] were added to resuspend the pellet. Finally, 12.5 μL each of H_2_O and 4× sample buffer (SB) [0.2 M Tris-HCl (pH 6.8), 8% SDS, 0.2% bromophenol blue, 20% glycerol, 10% beta-mercaptoethanol] were added. The final suspension was used for western blot analysis.

### Fluorescence activated cell sorting (FACS)

MONO-MAC-6 cells (2 × 10^6 ^cells/experiment) filtered by the LeukoCatch were stained by the CycleTEST™ PLUS DNA Reagent Kit (BD Bioscience) according to the manufacturer's instructions. Samples were divided into four tubes. Cells were counted four times for each sample to obtain the mean value using a FACScalibur with CellQuest software. This experiment was independently repeated twice.

### Western blot analysis and antibodies

For western blot analysis, the cell extracts were boiled, separated by 8% or 10% SDS-PAGE, and transferred to a polyvinylidine difluoride (PVDF) filter (Millipore, Bedford, MA). The filters were incubated with rabbit polyclonal antibodies specific for ERK1/2 (Cell Signaling Technology, Danvers, MA), GAPDH (Fitzgerald Industries International, Concord, MA), hemoglobin (MBL, Nagoya, Japan) FCN1 (Atlas Antibodies AB, Sweden), CD14 (Abcam, Cambridge, UK) or CD11a (Abcam). Then, the filter was incubated with horseradish peroxidase-conjugated anti-rat IgG (Amersham, Piscataway, NJ). Immunoreactive protein bands were visualized using Renaissance™ chemiluminescence reagents (DuPont NEN, Boston, MA).

### Application of LeukoCatch to blood samples

Blood samples (2 mL per tube) drawn from one of the authors (H.N, male, age 58) into EDTA vacuum blood collection tubes were sucked into the LeukoCatch syringe and poured back into the tube. This process was repeated for various numbers of times. Phosphate-buffered saline (5 mL) in a fresh container was sucked into the LeukoCatch syringe and poured back into the container. This process was repeated various times. Elution buffer (0.5 mL) was sucked into the LeukoCatch syringe and the dissolved protein extract was poured back to the tube. The protein extract in the flow-through fraction was either kept frozen at -80°C after addition of glycerol (20% final) for long-term storage, or was stored at -20°C after addition of 4× SB for western blot analysis.

### Hematology analysis of blood samples

The number of RBCs, platelets (PLTs), and WBCs in the fresh blood samples treated with or without LeukoCatch immediately after the blood was drawn were counted with an LH 750 hematology analyzer (Beckman Coulter, Fullerton, CA). Triplicate samples were counted.

### Preparation of PBMC extract by standard method

Fresh venous blood (2-4 mL) in EDTA (as an anticoagulant) was mixed with an equal volume of 2% dextran/saline solution and incubated at room temperature for 30 min to precipitate the RBCs. PBMCs in the supernatant were purified by density-gradient centrifugation on Percoll (density: 1.064 g/mL), and were subsequently suspended in SB for western blot analysis.

## Ethical permission

All of the experiments with use of samples of human blood were performed with the approval of the ethics committee of Osaka University.

## List of abbreviations

**FACS**: fluorescence activated cell sorting; **GAPDH**: glyceraldehyde-3-phosphate dehydrogenase; **LY**: lymphocyte; **MO**: monocyte; **NE**: neutrophil; **PBMC**: peripheral blood mononuclear cells; **PBS**: phosphate-buffered saline; **PVDF**: polyvinylidine difluoride; **PTL**: platelet; **RBC**: red blood cell; **SB**: sample buffer; **WBC**: white blood cell

## Competing interests

The authors declare that they have no competing interests.

## Authors' contributions

HN made contributions to conception and design of this study and drafted the manuscript. SK constructed the LeukoCatch devise. DO performed the biochemical study. NY contributed to tissue culture experiments. TO participated in the blood fractionation and data analysis. All authors read and approved the final manuscript.

## Pre-publication history

The pre-publication history for this paper can be accessed here:

http://www.biomedcentral.com/1472-6890/11/9/prepub
